# Electronic Health Behaviors Among US Adults With Chronic Disease: Cross-Sectional Survey

**DOI:** 10.2196/11240

**Published:** 2019-03-05

**Authors:** Lillian Madrigal, Cam Escoffery

**Affiliations:** 1 Rollins School of Public Health Emory University Atlanta, GA United States

**Keywords:** information seeking behavior, chronic disease, health promotion, eHealth, mobile apps, patient portals

## Abstract

**Background:**

With increased access to technology and the internet, there are many opportunities for utilizing electronic health (eHealth), internet, or technology-delivered health services and information for the prevention and management of chronic diseases.

**Objective:**

The aim of this paper was to explore (1) the differences in technology use, (2) Web-based health information seeking and use behaviors, (3) attitudes toward seeking health information on the Web, and (4) the level of eHealth literacy between adults aged 18 and 64 years with and without chronic disease.

**Methods:**

A cross-sectional internet survey was conducted in March 2017 with 401 US adults. Participant responses were examined to understand associations between chronic disease status and eHealth behaviors such as internet health-seeking behaviors and Web-based behaviors related to health, tracking health indicators with a mobile app, patient portal use, and preferences for health information.

**Results:**

About 1 in 3 (252/401, 37.2%) participants reported at least 1 chronic disease diagnosis. Seventy-five percent (301/401) of all participants reported having ever searched for health information on the Web. Participants with a chronic disease reported significantly higher instances of visiting and talking to a health care provider based on health information found on the Web (40.0% [48/120] vs 25.8% [46/178], χ^2^_2_=6.7; *P*=.01; 43.3% [52/120] vs 27.9% [50/179]; χ^2^_2_=7.6; *P*=.006). The uses of health information found on the Web also significantly differed between participants with and without chronic diseases in affecting a decision about how to treat an illness or condition (49.2% [59/120] vs 35.0% [63/180], χ^2^_3_=6.7; *P*=.04), changing the way they cope with a chronic condition or manage pain (40.8% [49/120] vs 19.4% [35/180], χ^2^_2_=16.3; *P*<.001), and leading them to ask a doctor new questions or get a second opinion (37.5% [45/120] vs 19.6% [35/179], χ^2^_2_=11.8; *P*<.001). Chronic disease participants were significantly more likely to be tracking health indicators (43.9% [65/148] vs 28.3%, [71/251] χ^2^_2_=10.4; *P*=.006). In addition, participants with chronic disease diagnosis reported significantly higher rates of patient portal access (55.0% [82/149] vs 42.1% [106/252], χ^2^_2_=6.3; *P*=.01) and use (40.9% [61/149] vs 21.0% [53/252], χ^2^_2_=18.2; *P*<.001). Finally, both groups reported similar perceived skills in using the internet for health information on the eHealth Literacy Scale (eHEALS). The majority of participants responded positively when asked about the usefulness of health information and importance of accessing health resources on the Web.

**Conclusions:**

The high rates of reported information seeking and use of internet-based health technology among participants with chronic disease may reflect the uptake in eHealth to help manage chronic disease conditions. Health care providers and educators should continue to seek ways to interact and support patients in their management of chronic disease through eHealth platforms, including capitalizing on Web-based resources, patient portals, and mobile phone apps for disease education and monitoring.

## Introduction

### Background

Electronic health (eHealth) encompasses wearable devices that sync automatically to Web-based dashboards, eHealth records accessed through mobile apps, social media networks, where patients can share experiences 24/7, Web-based medical diagnosis search engines, videoconference meetings with health care providers miles away, and much more. eHealth is defined as “health services and information delivered or enhanced through the Internet and related technologies [1].” Today, more than ever, adults have access to all sorts of internet-based technologies that can assist them with their health promotion and medical care.

It is now common practice for individuals to use the internet to seek information about their health conditions [2,3]. Furthermore, 1 area, in particular, where there is great potential for eHealth is with chronic disease management. Chronic disease self-management is the ability of patients to handle living with a chronic illness, including symptoms, treatment, physical and social consequences, and lifestyle changes [4]. Components of disease self-management encompass medical, role, symptom, and lifestyle management [5,6]. There is also a growing number of Web-based and mobile phone apps tools to help individuals manage their conditions and communicate with their health care providers. There are apps that assist patients with blood pressure monitoring, checking medical records, encouraging daily exercise, and reminding patients to take their medications [7].

Health care providers and health systems can support self-management by discussing goals and progress on patients’ self-management behaviors, offering self-management education, and following up on self-management goals and behaviors [8,9]. These practices could be enhanced by technology-based support tools such as disease indicator tracking and/or Web-based offerings or apps that support patient education. In general, technologies like smartphones tend to be used by younger adults, whereas people with chronic disease tend to be older, leading to disparities in internet-based technology use between people with and without chronic disease [3,10]. However, with growing accessibility to the internet, Web-based patient portals, and smartphones, there is the potential to use eHealth to improve chronic disease management, cost, and outcomes, as well as reduce barriers to care because of issues of mobility and distance to health care providers.

### Objectives

The purpose of this paper is to explore the differences between those with and without chronic disease(s) in their technology use, Web-based health information seeking and use behaviors, attitudes toward seeking health information on the Web, and level of eHealth literacy. We aim to answer the research questions (1) What is the difference in the prevalence of participation in Web-based health-related activities between adults with and without a chronic disease? and (2) Are there differences in level of eHealth literacy, engagement in eHealth behaviors, and health information resource preferences between these 2 groups?

## Methods

### Sample and Design

Data for this study were collected in March 2017 from a 1-time, cross-sectional internet survey of US adults drawn from Lightspeed Research (Lightspeed), an international, Web-based consumer survey company that recruits respondents by opt-in emails through multiple methods. Potential panelists registered with a unique email address and completed an in-depth demographic registration. Potential participants were eligible if they were adults (older than 18 years), had internet access, spoke English, and lived in the US participant locations verified by internet protocol addresses. Eligible respondents were invited via email to take the survey using a Health Insurance Portability and Accountability Act compliant version of SurveyMonkey. All participants were asked to read an informed consent form and give passive consent by clicking to begin the survey. No personally identifiable information was requested in the survey. Participants who completed the survey were given points by Lightspeed. This study was approved by the Emory University Institutional Review Board. At the end of the month-long recruitment period, 403 participants completed the survey, but only 401 were analyzed for this study as 2 individuals did not answer the question about chronic disease status.

### Measures

The survey contained a total of 109 items. Participants were asked about ownership of different devices (ie, smartphones, tablets, and computers), internet access, frequency of internet use, as well as engagement of eHealth behaviors such as tracking of health indicators, use of mobile apps for health, health information-seeking behaviors, and other Web-based activities related to health. These questions were adapted from the Pew Health and Internet Surveys [11,12]. Participants were also asked about how they used health information (eg, had conversation with family, changed behavior, and made a decision about a condition).

Participant eHealth literacy was measured using the eHealth Literacy Scale (eHEALS), an 8-item self-reported measure of perceived eHealth literacy [13]. Participants rated their level of agreement with statements on their knowledge, comfort, and perceived skills at locating, evaluating, and applying eHealth information to health problems using a 5-point Likert-type scale (1=strongly disagree, 5=strongly agree, and range 8-40). Higher scores reflect greater perceived levels of eHealth literacy.

Next, the participants were asked about how useful the internet is in making decisions about their health and the importance of accessing resources on the internet; questions were adopted from the Health Information National Trends Survey [14]. Participants rated the extent to which they trust different sources of health information (ie, internet, television, and government agency) on a scale of 1=not at all to 4=a lot. Participants also indicated how they preferred to receive health information from different sources (ie, person, print, and website).

Finally, demographic information on gender, race, Hispanic origin, income and education level, employment status, chronic illness diagnosis, reading level, geographic location, and rurality of their residence in the United States were assessed. Important to this study is the chronic disease diagnosis item. This was a 2-part item, first asking, “Have you been diagnosed or treated by a professional for a chronic disease?,” and then prompting participants who responded “yes” to select all chronic diseases for which they have ever been treated or diagnosed. Due to various definitions of *chronic disease* [15], this study included a broad list of 14 common chronic diseases as well as an “other” option for participants to specify additional conditions. In addition, 403 participants completed the survey. Out of which, 2 participants did not respond to the chronic illness diagnosis question and were removed from the study analysis resulting in a total of 401 participants.

### Data Analyses

The collected data were downloaded from SurveyMonkey and analyzed in SAS software version 9 (SAS Institute Inc). Descriptive statistics were run and used to report the chronic disease prevalence and types, demographics of the participants, levels of technology ownership and use, health monitoring, information seeking, other eHealth behaviors, and eHealth literacy and attitudes about the internet. Difference among these eHealth seeking and use variables and having chronic disease were run through independent chi-square tests and independent *t* tests. Independent chi-square tests were calculated to compare the frequency of the categorical variables between those with and without chronic disease. Independent *t* tests were used for all continuous variables.

The range, mean score, and SD were calculated for the perceived eHealth literacy level. We computed a total score for the eHEALS and calculated a Cronbach alpha to measure reliability of the total scale for the total sample and by chronic disease status groups. We examined the difference between perceived levels of eHealth literacy and chronic disease status through independent sample *t* tests. The level of eHealth behavior engagement was also compared between chronic disease status groups. eHealth behaviors were split into 2 groups: “informational” and “participatory.” Informational eHealth behaviors are those that include seeking information on the Web, whereas participatory behaviors are those that include some kind of active engagement from the participant (eg, to post, share, or comment on health-related issues via social media, to join or develop Web-based health communities, or maintain healthy lifestyles) [16]. Finally, we compared eHEALS scores in both groups by eHealth behaviors using independent sample *t* tests. The level of significance for all tests was set at *P*<.05.

## Results

### Respondent Characteristics

About 1 in 3 (149/401, 37.2%) participants reported that they had been diagnosed with at least 1 chronic disease (see Table 1). Of the 37.2% of participants who reported a chronic disease diagnosis, the most frequently reported were high blood pressure (77/149, 51.7%), high cholesterol (60/149, 40.3%), and diabetes (48/149, 32.2%). The other chronic diseases commonly reported were arthritis, depression, asthma, anxiety, heart disease, and bronchitis. Both genders were represented at 50% and participants’ ages ranged from 18 to 90 years (mean 50.7, SD 17.1). Race reflected the racial diversity of the United States with 66.8% (268/401) white, 19.2% (77/401) black, and 14.0% (56/401) other races, and 19.5% (78/399) were of Hispanic origin. About 47% (190/400) of participants had a college degree or higher, 43.8% (175/400) were employed either full-time or part-time, and 36.8% (147/399) reported household incomes over US $75,000. Many of the participants were married (202/399, 50.6%) and lived in urban (136/398, 34.2%) or suburban (199/398, 50.0%) areas, from all regions in the United States.

There were significant differences in age, employment, household income, and marital status between the groups with and without chronic disease diagnosis. The average age was significantly higher in the chronic disease group (mean 57.5, SD 15.5) compared with (mean 46.6, SD 16.6) the no chronic disease group (*t*_401_=−6.49; *P*<.001). The chronic disease group was significantly less likely to be employed and contained a larger retired population (χ^2^_3_=14.2; *P*<.001), with a larger proportion of the chronic disease population making an income of less than US $25,000 (χ^2^_3_=8.3; *P*=.02). The chronic disease group had a significantly higher percentage of married individuals (χ^2^_3_=6.7; *P*=.04) than the group with no chronic diseases.

### Technology Ownership and Access

A large majority of participants owned laptops (288/401, 71.8%) and smartphones (288/401, 71.8%), followed by desktop computers, tablets, and digital versatile disc players (see Table 1). No chronic disease participants (190/252, 75.4%) reported owning a smartphone significantly more than chronic disease participants (98/149, 65.8%), (χ^2^_2_=4.3; *P*=.04). Almost all participants reported having access to a computer (398/400, 99.3%) and using the internet several times a day (205/401, 51.1%) or almost constantly (148/401, 36.9%).

**Table 1:** Participant characteristics.

Characteristic		Chronic disease	No chronic disease	Total	*P* value
Chronic disease diagnosis, n (%)		149 (37.2)	252 (62.8)	401 (100)	—^a^
**Type of chronic disease, n (%)**					
	High blood pressure	77 (51.7)	—	—	—
	High cholesterol	60 (40.3)	—	—	—
	Diabetes	48 (32.2)	—	—	—
	Arthritis	38 (25.5)	—	—	—
	Depression	27 (18.1)	—	—	—
	Asthma	20 (13.4)	—	—	—
	Anxiety	18 (12.1)	—	—	—
	Heart disease	13 (8.7)	—	—	—
	Bronchitis	12 (8.1)	—	—	—
	Other chronic disease	64 (43.0)	—	—	—
Age (years), mean (SD)		57.5 (15.5)	46.6 (16.6)	50.7 (17.1)	<.001
**Gender (n=399), n (%)**					.49
	Male	78 (52.3)	120 (47.6)	198 (49.4)	—
	Female	70 (47.0)	130 (51.6)	200 (49.9)	—
	Other	—	1 (0.4)	1 (0.2)	—
**Race (N=401), n (%)**					.87
	White	102 (68.5)	166 (65.9)	268 (66.8)	—
	Black	27 (18.1)	50 (19.8)	77 (19.2)	—
	Other	20 (13.4)	36 (14.3)	56 (14.0)	—
	Hispanic origin (n=399)	30 (20.1)	48 (19.2)	78 (19.5)	.82
**Level of school (n=400), n (%)**					.43
	High school graduate, GED^b^, or less	24 (16.1)	49 (19.5)	73 (18.3)	—
	Some college	58 (38.9)	79 (31.5)	137 (34.3)	—
	College	42 (28.2)	82 (32.7)	124 (31.0)	—
	Graduate	25 (16.8)	41 (16.3)	66 (16.5)	—
**Employment (n=400), n (%)**					<.001
	Employed, full-time or part-time	52 (35.1)	123 (48.8)	175 (43.8)	—
	Retired	54 (36.5)	50 (19.8)	104 (26.0)	—
	Other	42 (28.4)	79 (31.3)	121 (30.3)	—
**Household income is US $ (n=399), n (%)**					.02
	Less than 24,999	43 (29.3)	45 (17.9)	88 (22.1)	—
	25,000-74,999	50 (34.0)	114 (45.2)	164 (41.1)	—
	75,000 or more	54 (36.7)	93 (36.9)	147 (36.8)	—
**Marital status (n=399), n (%)**					.04
	Married	82 (55.0)	120 (48.0)	202 (50.6)	—
	Divorced or separated or widowed	28 (18.8)	34 (13.6)	62 (15.5)	—
	Single	39 (26.2)	96 (38.4)	135 (33.8)	—
**Urban-rural location (n=398), n (%)**					.08
	Urban	52 (35.6)	84 (33.3)	136 (34.2)	—
	Suburban	64 (43.8)	135 (53.6)	199 (50.0)	—
	Rural	30 (20.5)	33 (13.1)	63 (15.8)	—
**US geography (n=398), n (%)**					.56
	Northeast	29 (19.6)	64 (25.6)	93 (23.4)	—
	South	36 (24.3)	62 (24.8)	98 (24.6)	—
	Midwest	31 (20.9)	39 (15.6)	70 (17.6)	—
	Southwest	16 (10.8)	27 (10.8)	43 (10.8)	—
	West	36 (24.3)	58 (23.2)	94 (23.6)	—
**Technology ownership (N=401), n (%)**					—
	Laptop	105 (70.5)	183 (72.6)	288 (71.8)	.64
	Smartphone	98 (65.8)	190 (75.4)	288 (71.8)	.04
	Desktop computer	96 (64.4)	138 (54.8)	234 (58.4)	.06
	Tablet (Kindle, iPad)	79 (53.0)	148 (58.7)	227 (56.6)	.26
	Digital versatile disk or Blu ray player	81 (54.4)	139 (55.2)	220 (54.9)	.88
	Game console	46 (30.9)	98 (38.9)	144 (35.9)	.11
	Cell phone	47 (31.5)	57 (22.6)	104 (25.9)	.05
Internet access (n=400), n (%)		149 (100.0)	249 (98.8)	398 (99.3)	.18
**Frequency of internet use (N=401), n (%)**					.27
	Almost constantly	48 (32.2)	100 (39.7)	148 (36.9)	—
	Several times a day	80 (53.7)	125 (49.6)	205 (51.1)	—
	About once a day to less often	21 (14.1)	27 (10.7)	48 (12.0)	—

^a^Not applicable.

^b^GED: General Educational Development.

### Health Information Seeking and Technology Use for Health

Many of the participants (301/401, 75.1%) reported having ever searched for health information on the internet and to have searched for health information on the Web in the past month (172/401, 42.9%; mean 3.1, SD 8.8). The top 4 search topics were diet/nutrition, exercises, medicines, and quick remedies. Participants with chronic diseases were significantly more likely to have searched about medicines than the no chronic disease participants (25.5% [38/149] vs 13.5% [34/252], *t*_401_=3.06; *P*=.02).

About 34.1% of participants reported tracking any health indicator regularly (see Table 2). Chronic disease participants were significantly more likely to be tracking health indicators (43.9% [65/148] vs 28.3% [71/251], χ^2^_2_=10.4; *P*=.006). Participants reported tracking health indicators by keeping track in their head (82/401, 20.4%), on paper (62/401, 15.5%), with a phone app (56/401, 14.0%), medical device (48/401, 12.0%), website (38/401, 9.5%), wearable device (36/401, 9.0%), and/or computer program (33/401, 8.2%). Of the 24.2% (97/401) of participants who reported having health focused mobile phone apps, the top apps were related to exercise (73/401, 18.2%), diet (35/401, 8.7%), and weight (25/401, 6.2%). There were no meaningful differences between chronic disease and no chronic disease groups.

Other common health activities on the internet included reading about someone else’s health experience (40.3% [60/149] chronic disease group vs 33.3% [84/252] no chronic disease group), watching a video about health (34.9% [52/149] vs 28.1% [70/252]), and surfing the Web to find others who have similar health conditions (27.5% [41/149] vs 24.2% [61/252]). Chronic disease participants reported significantly higher activity related to signing up for health email updates (29.5% [44/149] vs 19.8% [50/252]; χ^2^_2_=5.3; *P*=.02) and downloading health insurance forms or applying for health insurance (25.2% [37/147] vs 14.7% [37/252], χ^2^_2_=6.8; *P*=.009). Participants used the information they found on the Web in a variety of ways. Participants with chronic disease reported significantly higher instances of visiting a health care provider based on health information found on the Web (40.0% [48/120] vs 25.8% [46/178], χ^2^_2_=6.7; *P*=.01) and talking with a provider about health information found on the Web (43.3% [52/120] vs 27.9% [50/179], χ^2^_2_=7.6; *P*=.006) than those with no chronic diseases. The uses of health information found on the Web also significantly differed between participants with and without chronic diseases in affecting a decision about how to treat an illness or condition (49.2% [59/120] vs 35.0% [63/180], χ^2^_3_=6.7; *P*=.04), changing the way they cope with a chronic condition or manage pain (40.8% [49/120] vs 19.4% [35/180], χ^2^_2_=16.3; *P*<.001), and leading them to ask a doctor new questions or get a second opinion (37.5% [45/120] vs 19.6% [35/179], χ^2^_2_=11.8; *P*<.001).

Finally, related to health system portal use, 46.9% (188/401) of participants reported having access to a patient portal or app with 28.4% (114/401) of participants who have ever used a patient portal in the last 12 months. Patients with a chronic disease diagnosis reported significantly higher rates of patient portal access (55.0% [82/149] vs 42.1% [106/252], χ^2^_2_=6.3; *P*=.01) and patient portal use in the last 12 months (40.9% [61/149] vs 21.0% [53/252], χ^2^_2_=18.2; *P*<.001). Of those with access to a patient portal, frequent uses included viewing test or lab results (89/188, 47.3%), emailing the doctor or doctor’s office (73/188, 38.8%), and setting up an appointment on the Web (59/188, 31.4%). There were no meaningful differences in patient portal activities between those with or without a chronic disease diagnosis.

### Electronic Health Literacy

Generally, both groups, those with and without chronic disease, reported similar perceived skills in using the internet for health information. The total eHEALS score of the chronic disease group was significantly higher by 1.23 points (*t*_393_=−1.99; *P*=.03, see Table 3) than that of the group with no chronic diseases. The average total score for all participants was 29.89 (SD 5.95); it was 30.66 (SD 6.10) for those with chronic disease and 29.43 (SD 5.81) for those without chronic disease. The chronic disease participants generally reported slightly higher confidence in 7 out of the 8 eHEALS items. The scale had high internal consistency with a Cronbach alpha of .936.

Overall, both groups reported neutral to positive feelings regarding the usefulness of the internet in making decisions about health (mean 3.65, SD 0.98) as well as the importance of accessing health resources on the internet (mean 3.69, SD 1.07). Participants with chronic disease significantly reported slightly higher levels of confidence that they could get advice or information about health or medical topics if needed (mean 2.26 vs mean 2.48, *t*_399_=2.23; *P*=.03).

### Electronic Health Behavior Engagement and Electronic Health Literacy Rate Scale Scores

There were no significant differences in participant engagement in eHealth behaviors (Table 4). eHealth behaviors were split into 2 groups: “informational” and “participatory.” Informational eHealth behaviors are those that include seeking information on the Web, whereas participatory behaviors are those that include some kind of active engagement from the participant (eg, to post, share, or comment on health-related issues via social media, to join or develop Web-based health communities, or maintain healthy lifestyles) [16]. The list of eHealth behaviors by type is included in Table 5. On average, the chronic disease group reported engaging in 4.57 different eHealth behaviors (SD 3.32), whereas the no chronic disease group reported engaging in 3.97 eHealth behaviors (SD 3.22). Both groups reported higher engagement in informational eHealth behaviors compared with participatory ones. This echoes the results described in Table 2.

**Table 2:** Participant health information–seeking and technology use for health behaviors.

Item		Chronic disease, n (%)	No chronic, disease n (%)	Total, N (%)	*P* value
Ever looked on the Web for health info (N=401)		120 (80.5)	181 (71.8)	301 (75.1)	.05
Looked on the Web for health info in the past month (N=401)		62 (41.6)	110 (43.7)	172 (42.9)	.69
Average number of times in past month looked for health information (n=297)		4.06 (SD 12.94)	2.43 (SD 4.05)	3.08 (SD 8.79)	.12
Do you track a health indicator? (n=399)		65 (43.9)	71 (28.3)	136 (34.1)	.006
**Tracking method (N=401)**					
	In their head	34 (22.8)	48 (19.0)	82 (20.4)	.37
	Paper	24 (16.1)	38 (15.1)	62 (15.5)	.78
	App on phone	19 (12.8)	37 (14.7)	56 (14.0)	.59
	Medical device	33 (22.1)	15 (6.0)	48 (12.0)	<.001
	Website or on the Web	14 (9.4)	24 (9.5)	38 (9.5)	.97
	Wearable device	6 (4.0)	30 (11.9)	36 (9.0)	.008
	Computer program	17 (11.4)	16 (6.3)	33 (8.2)	.07
Do you use any health apps? (N=401)		36 (24.2)	61 (24.2)	97 (24.2)	>.99
**Types of health apps used (N=401)**					
	Exercise	25 (16.8)	48 (19.0)	73 (18.2)	.57
	Diet, food, calorie counter	13 (8.7)	22 (8.7)	35 (8.7)	>.99
	Weight	7 (4.7)	18 (7.1)	25 (6.2)	.33
	Blood pressure	10 (6.7)	9 (3.6)	19 (4.7)	.15
	WedMD or health organization	11 (7.4)	12 (4.8)	23 (5.7)	.28
	Menstrual cycle	8 (5.4)	7 (2.8)	15 (3.7)	.19
	Sleep	1 (0.7)	14 (5.6)	15 (3.7)	.01
	Blood sugar or diabetes	8 (5.4)	5 (2.0)	13 (3.2)	.06
	Medication management	7 (4.7)	2 (0.8)	9 (2.2)	.02
	Mood or feelings	5 (3.4)	2 (0.8)	7 (1.7)	.06
**Health topics searched in the past month (N=401)**					
	Diet or nutrition	39 (26.2)	56 (22.2)	95 (23.7)	.37
	Exercise	19 (12.8)	63 (25.0)	82 (20.4)	.003
	Medicines	38 (25.5)	34 (13.5)	72 (18.0)	.002
	Quick remedy	25 (16.8)	26 (10.3)	51 (12.7)	.06
**Other health activities on the internet (N=401)**					
	Read someone else’s commentary or experience about health issues	60 (40.3)	84 (33.3)	144 (35.9)	.16
	Watched a video about health	52 (34.9)	70 (28.1)	122 (30.7)	.16
	Gone on the Web to find others who might have health concerns similar to you	41 (27.5)	61 (24.2)	102 (25.4)	.46
	Signed up to receive email updates	44 (29.5)	50 (19.8)	94 (23.6)	.02
	Download forms or applied for health insurance on the Web	37 (25.2)	37 (14.7)	74 (18.5)	.009
**Uses of health information (n=299)**					
	Have a conversation with friend or family member	46 (38.3)	77 (43.0)	123 (41.1)	.42
	Changed behavior	23 (19.2)	41 (23.0)	64 (21.5)	.43
	Made a decision about condition	50 (41.7)	68 (38.0)	118 (39.5)	.52
	Visited a doctor or provider	48 (40.0)	46 (25.8)	94 (31.5)	.01
	Talked with a doctor or provider	52 (43.3)	50 (27.9)	102 (34.1)	.006
**Did the information found on the Web... (n=301)**					
	Affect a decision about how to treat an illness or condition	59 (49.2)	63 (35.0)	122 (40.5)	.04
	Change your overall approach to maintaining your health or someone else’s health	40 (33.3)	55 (30.6)	95 (31.6)	.59
	Change the way you cope with a chronic condition or manage pain	49 (40.8)	35 (19.4)	84 (27.9)	<.001
	Affect a decision about whether to see a doctor	36 (30.0)	46 (25.6)	82 (27.2)	.40
	Lead you to ask a doctor new questions, or get a second opinion	45 (37.5)	35 (19.6)	80 (26.8)	<.001
Doctor has recommended a particular health or medical website to you? (n=399)		23 (15.5)	13 (5.2)	36 (9.0)	<.001
**Health system portal use (N=401)**					
	Number of people with patient portal access (portal or app)	82 (55.0)	106 (42.1)	188 (46.9)	.01
	People who have used the portal in the last 12 months	61 (40.9)	53 (21.0)	114 (28.4)	<.001
**Uses of the patient portal for those with access (n=188)**					
	Email your doctor or office	28 (34.1)	45 (42.5)	73 (38.8)	.24
	Set an appointment on the Web	17 (20.7)	42 (39.6)	59 (31.4)	.01
	See tests or laboratory results	36 (43.9)	53 (50.0)	89 (47.3)	.41

**Table 3:** Participant health literacy and perceptions of internet health resources. Cronbach alpha: chronic disease=.940; no chronic disease=.933; total=.936.

eHEALS^a^ item^b^	Chronic disease, mean (SD)	No chronic disease, mean (SD)	Total, mean (SD)	*P* value
I know how to find helpful health resources on the internet (n=399)	4.08 (0.78)	3.74 (0.89)	3.87 (0.86)	<.001
I know how to use the internet to answer my health questions (N=401)	3.94 (0.84)	3.81 (0.82)	3.86 (0.83)	.13
I know what health resources are available on the internet (n=400)	3.64 (0.97)	3.67 (0.84)	3.66 (0.89)	.73
I know where to find helpful health resources on the internet (n=399)	3.93 (0.90)	3.78 (0.86)	3.83 (0.88)	.10
I know how to use health information I find on the internet to help me (N=401)	3.92 (0.92)	3.80 (0.88)	3.85 (0.90)	.20
I have the skills I need to evaluate the health resources I find on the internet (n=400)	3.82 (0.94)	3.69 (0.89)	3.74 (0.91)	.17
I can tell high quality from low quality health resources on the internet (n=399)	3.69 (0.95)	3.48 (0.91)	3.56 (0.93)	.03
I feel confident in using information from internet to make health decision (n=399)	3.61 (0.94)	3.52 (0.94)	3.55 (0.94)	.33
Total eHEALS score (n=393)	30.66 (6.10)	29.43 (5.81)	29.89 (5.95)	.05
Usefulness of internet to making decisions about health^c^ (n=400)	3.68 (1.01)	3.62 (0.95)	3.65 (0.98)	.53
Importance of accessing health resources on the internet^d^ (n=399)	3.72 (1.17)	3.67 (1.01)	3.69 (1.07)	.68
Overall, how confident are you that you could get advice or information about health or medical topics if you needed it?^e^ (N=401)	2.26 (1.00)	2.48 (0.99)	2.40 (1.00)	.03

^a^eHEALS: electronic health literacy scale.

^b^1=strongly disagree to 5=strongly agree.

^c^1=not useful at all to 5=very useful.

^d^1=not important at all to 4=very important.

^e^1=not confident at all to 5=completely confident.

**Table 4:** eHealth^a^ behavior engagement.

Type of eHealth behavior	eHealth behaviors participants reported engaging in		*P* value
	Chronic disease (n=149), mean (SD)	No chronic disease (n=252), mean (SD)	
eHealth behaviors	4.57 (3.32)	3.97 (3.22)	.07
Informational eHealth behaviors	3.0 (2.06)	2.7 (2.07)	.11
Participatory eHealth behaviors	1.6 (1.55)	1.3 (1.49)	.12

^a^eHealth: electronic health.

Higher health literacy eHEALS scores were associated with engaging in different eHealth behaviors (Table 5). In Table 5, for both the chronic disease and no chronic disease groups, average eHEALS scores were compared among participants based on their eHealth engagement behavior. For all informational eHealth behaviors for both chronic disease status groups, there were significantly higher eHEALS scores in the group that reported engaging in the behavior. With the participatory eHealth behaviors, the average eHEALS scores were also higher in those that engaged in the behavior versus those that did not (eg, tracking health indicators using a mobile app, patient portal use in last 12 months). However, the statistical significance varied. One reason for this could be the overall lower reported engagement in the participatory eHealth behaviors.

**Table 5:** Comparison of eHealth^a^ literacy scale scores by eHealth behavior engagement.

eHealth behavior		Chronic disease group					No chronic disease group				
		Those engaging in eHealth behavior		Those not engaging in eHealth behavior		*P* value	Those engaging in eHealth behavior		Those not engaging in eHealth behavior		*P* value
		Average eHEALS^b^ score	n (%)	Average eHEALS score	n (%)		Average eHEALS score	n (%)	Average eHEALS score	n (%)	
**Informational eHealth behaviors**											
	Web-based health information seeking (ever)	31.29	119 (80.4)	28.03	29 (19.6)	.009	30.70	178 (72.7)	26.04	67 (27.3)	<.001
	Web-based health information seeking (in the last month)	31.97	87 (58.8)	28.79	61 (41.2)	.002	31.26	139 (56.7)	27.03	106 (43.3)	<.001
	Web-based information seeking on the phone (ever)	32.82	45 (30.6)	29.76	102 (69.4)	.005	31.31	87 (35.5)	28.39	158 (64.5)	<.001
	Signed up for health email updates or alerts	32.25	44 (29.9)	30.01	103 (70.1)	.04	31.71	49 (20.0)	28.86	196 (80.0)	.002
	Went on the Web to read about other’s experiences	31.93	60 (40.5)	29.78	88 (59.5)	.04	31.33	82 (33.5)	28.47	163 (66.5)	<.001
	Went on the Web to watch health-related videos	32.54	52 (35.1)	29.64	96 (64.9)	.005	31.84	69 (28.4)	28.41	174 (71.6)	<.001
	Went on the Web to find others with similar health issues	32.50	40 (27.0)	29.97	108 (73.0)	.03	31.62	60 (24.5)	28.72	185 (75.5)	<.001
**Participatory eHealth behaviors**											
	Download forms or applied for health insurance on the Web	32.49	37 (25.2)	30.10	110 (74.8)	.04	30.92	36 (14.7)	29.17	209 (85.3)	.10
	Web-based tracking of weight or diet indicators	33.41	22 (14.9)	30.17	126 (85.1)	.02	30.69	48 (19.6)	29.12	197 (80.4)	.09
	Web-based tracking of other health indicators	34.17	30 (20.4)	29.68	117 (79.6)	<.001	31.55	33 (13.5)	29.12	211 (86.5)	.03
	Posted on social networking	32.39	28 (18.9)	30.25	120 (81.1)	.09	31.27	41 (16.7)	29.06	204 (83.3)	.03
	Posted on a Web-based discussion group	30.94	18 (12.2)	30.56	129 (87.8)	.80	31.45	20 (8.2)	29.25	225 (91.8)	.11
	Tracking health indicators using a mobile app	32.29	35 (23.6)	30.15	113 (76.4)	.07	31.15	60 (24.5)	28.87	185 (75.5)	.008
	Patient portal use in last 12 months	31.48	60 (40.5)	30.09	88 (59.5)	.17	31.75	83 (33.9)	28.24	162 (66.1)	<.001

^a^eHealth: electronic health.

^b^eHEALS: eHealth Literacy Scale.

**Table 6:** Distribution of participants’ preferences for methods of receipt of electronic health information: "In which of these ways would you like to get information and advice about how to manage health conditions and make changes in health behaviors (diet, exercise)?"

Communication method	Chronic disease, n	No chronic disease, n	Total, N (%)
Get information from internet websites	58	87	145 (36.2)
Print materials (eg, brochures, tip sheets)	65	54	119 (29.7)
Health newsletters or information by email	49	57	106 (26.4)
In-person counseling with a patient educator	43	51	94 (23.4)
Communications using doctor’s secure email	33	37	70 (17.5)
Get information from your doctor’s home page or patient portal	30	32	62 (15.5)
Telephone sessions with a health coach or educator or provider	33	23	56 (14.0)
Health newsletters or information by mail	29	23	52 (13.0)
Watch Web-based videos on doctor's website, and YouTube	22	29	51 (12.7)
Use a health app on your tablet or smartphone	21	29	50 (12.5)

### Channels for Health Information

Finally, the top 10 sources in which participants preferred to receive health information included via the internet as the top choice (58/401, 36.2%), followed by print materials (65/401, 29.7%), health newsletters or information via email (49/401, 26.4%), in-person counseling with a patient educator (43/401, 23.4%), and direct communication with the doctor via email (33/401, 17.5%; Table 6). There were no significant differences in methods for receiving health information between the 2 groups.

## Discussion

### Principal Findings

Overall, this study found that adults with and without a chronic disease diagnosis are going to the internet to seek health information. Participants with chronic disease appear to be slightly more activated in their eHealth behaviors in searching for health information, tracking health indicators, and using a patient portal. A number of other studies have also reported increased eHealth behaviors for people with chronic disease; these eHealth behaviors include looking for information regarding their conditions [8,17,18], using Web-based or computer tools to help manage their conditions [19-21], and using portal platforms to increase engagement in their own personal health information as well as increase communication with their health care providers [22,23].

The differences in the study population characteristics between the 2 groups reflect the national prevalence of chronic diseases in older populations [24]. The chronic diseases commonly reported by those who have been diagnosed with at least 1 chronic disease in our sample also reflect the most prevalent chronic diseases in the United States [24]. Study participants with chronic disease tended to be older, retired, at lower income levels, and married. These characteristics could influence their eHealth behaviors. For example, older adults do not typically use as much technology compared with younger populations [25]. This study also found some examples of this technology gap (eg, less smartphone ownership); however, it was not great and did not result in huge differences in eHealth behaviors. Predictors of eHealth behaviors within this population are discussed in a previously published paper [26]. The authors found that, generally, younger age, being female, and higher eHEALS scores were significant predictors of looking on the Web for health information in the past month and having a health-related mobile app.

This study’s results indicate high access to the internet and eHealth resources. In 2010 and 2013, national survey data from the Pew Research Center found that people with chronic diseases were less likely to have internet access [8,18]. In this study, there were no differences between groups in access or frequency of internet use, probably because of the rise in ubiquitous internet access and use in recent years. In a 2018 national report by Pew, 89% of US adults reported regular access to the internet [27]. In addition, across all participants, the top most preferred way (36.2%) to receive health information was via the internet (see Table 6). Consistent with the other literature, eHealth, that is health services and information delivered or enhanced through the internet and related technologies, is currently the norm and most common way through which individuals seek health information in the United States [7,18,21,28]. Similar to the previous Pew studies, we also found that those with chronic conditions were more likely than other adults to engage in certain eHealth-related behaviors, such as tracking a health indicator or following up with a medical professional based on information they found on the Web about their condition [7,8]. However, there were no significant differences in the average number of eHealth behaviors in which participants reported engaging between those with and without a chronic disease (see Table 4). These findings demonstrate a consistency in technology-based behaviors for health among adults.

The use of eHealth resources requires eHealth literacy, “the ability to read, use computers, search for information, understand health information, and put it into context [13].” This study found moderately high rates of health literacy among all participants using the eHEALS (mean 29.89 out of a possible 40 points). The eHEALS has been tested for validity and reliability in many populations, including those with chronic disease [13,17,29]. In addition, we found that higher eHEALS average scores were associated with engagement in eHealth behaviors, particularly with information-seeking behaviors. Although eHealth behaviors have become commonplace, future interventions may be necessary to improve literacy and capacity to use eHealth for the community to impact its disease management and/or well-being. Stellesfon et al explored levels of health literacy among people with chronic disease using the eHEALS and found that although participants reported moderate levels of eHealth literacy, they were not as confident in the ability to distinguish the quality of the health information found on the Web. The participants in this study within both groups also reported lower confidence in high- versus low-quality Web-based health information.

Beyond seeking health information, the rise of Web-based technology has allowed for greater management of personal health information, especially through patient portals. Coughlin et al define patient portals as “Web-based, patient-centered health care information systems linked to a patient’s electronic medical record.” Patient portals have many functions including, but not limited to, the ability to communicate with health care providers, checking health records and lab results, requesting prescription refills, viewing educational materials, and scheduling appointments [30]. Since the 2009 US Health Information Technology for Economic and Clinical Health Act, health care providers and systems have dramatically increased the availability of electronic health records and access to patient portals [31]. In 2014, the Office of the National Coordinator for Health Information Technology reported that about 40% of US adults have access to their electronic health records via Web-based patient portals and around 55% of those adults have actually gone on the Web to access them [32]. In this study, approximately half (188/401, 46.9%) of the participants reported having access to a patient portal, and of those with access, 60.1% (114/188) reported using the patient portal in the last 12 months.

Since 2009, patient portals have been studied extensively to understand current access and usage trends, patient and provider attitudes toward patient portals, and the benefits of patient portals. However, as uptake increases, more research is still needed in this area, especially as it pertains to the management of chronic diseases. In the Coughlin et al review, several studies found benefits to using health care system patient portals to assist patients manage their chronic disease(s) and improve patient outcomes. Other studies focused on patients with chronic disease have reported that portals have the ability to improve access and communication with health care providers [22,23]. Not only do the patients have a direct way to message their health care provider, but having access to their own health data and knowing that their provider has their complete health history allow for patients to discuss concerns in more detail during their visits. This study’s participants with chronic disease had higher access and use of patient portals than the no chronic disease group. Participants used the patient portal mostly for viewing test or lab results (89/188, 47.3%), emailing the doctor or doctor’s office (73/188, 38.8%), and setting up an appointment on the Web (59/188, 31.4%). These results support previous findings that patient portals are a good potential avenue for intervention with this population and are enhancing the way patients interact with their providers as well as understand their own health data. This portal technology has the potential to increase the efficiency and quality of health care; however, much more research is needed to rigorously test the benefits and increase both provider and patient uptake of these systems [23]. Continued research is necessary to better understand the purpose of portal use, frequency of use, and the relationship between use and better health outcomes.

Another key eHealth behavior regarding personal health information has been the tracking and management of health indicators. With the rise of wearable and smartphone technologies, there are many more ways for people to collect and record data on their own health behaviors and outcomes. A 2012 survey conducted by the Pew Research Center’s Internet and American Life Project on mobile health behaviors found that 69% of US adults kept track of at least 1 health indicator (ie, weight, diet, exercise routine, or symptom). They also found that people living with 1 or more chronic conditions were no more likely than other US adults to track their weight, diet, or exercise routine, but are more likely to track disease-related health indicators (eg, blood pressure). Their study found that 49% of trackers say they keep track of progress “in their heads,” 34% say they track the data on paper, like in a notebook or journal, and 21% say they use some form of technology to track their health data [33]. In this study, only 34.1% (136/399) of participants reported tracking any health indicator regularly, which is lower. Chronic disease participants were significantly more likely to be tracking health indicators and participants similarly reported the same top 3 tracking methods (in their head, on paper, with some form of technology). In 2012, Pew Research Center also reported that 19% of smartphone owners have at least 1 health app on their phone [33]. Recently, with the increased prevalence of smartphone technology, we expected to see much higher rates of use of smartphone apps to track health indicators, but only 24.2% (97/401) of the study participants reported having any health-focused smartphone apps. These results may be partially because our broader sample of different age groups that may differ from other research in this area. This is a critical area of chronic disease management for patients to track important health indicators (ie, weight, A1c, blood pressure, and steps for physical activity) and share relevant data to their providers as necessary for their health management. Health apps and passive technology such as wearable devices can assist patients with these tasks [34].

Recent studies that have examined mobile app use within chronic disease populations have found that although a wide offering of chronic disease management apps is on the market, they are lacking in their functionality, clinical utility, and usability [35]. In 2018, Escoffery and colleagues came to similar conclusions after reviewing apps for epilepsy self-management. They found that although a number of apps existed, many were very limited, and there was a lack of theory and evidence-grounded health behavior techniques applied to these apps to assist with chronic disease management [36]. Other studies have found variability in app design and education and indicated the need for detailed education and self-management features [37]. In addition to this lack of quality, there is a lack of use by those who are at highest risk or poorest health status and behaviors [38]. Much further research is needed in the development, testing, and accessibility of quality mobile health apps for people with chronic diseases focused on education, monitoring of health status, social support, and promotion of self-management.

### Limitations

Limitations of this study include that the sample may not be representative of all US adults. To reduce this selection bias, we attempted to recruit a cross-section of the population across gender, racial, ethnic, and educational groups. In addition, as the survey was conducted via a Web-based platform, there is a bias toward individuals with access to and familiar with the internet. However, our finding is consistent with another national study with approximately 89% of US adults with regular access to the internet and their high frequency of internet use (26% reporting that they are almost constantly on the Web) [27]. Reporting bias because of self-reported data is another limitation, especially for our key variable of interest: chronic disease status. Our survey took a broader approach to defining chronic disease and simply asked participants to self-report if they have ever been diagnosed or treated for a chronic disease. We did not medically verify disease diagnosis. In addition, the sample size is smaller than similar survey studies [10,38,39] and comparisons between chronic disease and no chronic disease groups are limited by the small numbers. Finally, the eHEALS only includes skills related to certain eHealth behaviors and does not yet include eHealth behaviors relate to mobile apps and social media.

### Conclusions

This study presents current technology ownership, eHealth literacy, information seeking, eHealth behaviors, and impacts of eHealth information seeking among a sample of US adults with chronic diseases. The observed high information seeking and use of internet-based health technology among participants with chronic disease may reflect uptake in eHealth to help manage chronic disease conditions. Health care providers and educators should continue to seek ways to interact and support patients in their management of chronic disease through eHealth platforms, including Web-based resources, patient portals, and smartphone apps for disease monitoring.

## Abbreviations

eHEALS: eHealth Literacy Scale
eHealth: electronic health
